# The unmet promise of trustworthy AI in healthcare: why we fail at clinical translation

**DOI:** 10.3389/fdgth.2024.1279629

**Published:** 2024-04-18

**Authors:** Valerie K. Bürger, Julia Amann, Cathrine K. T. Bui, Jana Fehr, Vince I. Madai

**Affiliations:** ^1^ QUEST Center for Responsible Research, Berlin Institute of Health (BIH), Charité—Universitätsmedizin Berlin, Berlin, Germany; ^2^ Strategy and Innovation, Careum Foundation, Zurich, Switzerland; ^3^ Digital Health & Machine Learning, Hasso Plattner Institute for Digital Engineering, Digital Engineering Faculty, University of Potsdam, Potsdam, Germany; ^4^ Faculty of Computing, Engineering, and the Built Environment, School of Computing and Digital Technology, Birmingham City University, Birmingham, United Kingdom

**Keywords:** AI, machine learning, ethics, trustworthiness, trust, translation, healthcare, medicine

## Abstract

Artificial intelligence (AI) has the potential to revolutionize healthcare, for example via decision support systems, computer vision approaches, or AI-based prevention tools. Initial results from AI applications in healthcare show promise but are rarely translated into clinical practice successfully and ethically. This occurs despite an abundance of “Trustworthy AI” guidelines. How can we explain the translational gaps of AI in healthcare? This paper offers a fresh perspective on this problem, showing that failing translation of healthcare AI markedly arises from a lack of an operational definition of “trust” and “trustworthiness”. This leads to (a) unintentional misuse concerning what trust (worthiness) is and (b) the risk of intentional abuse by industry stakeholders engaging in ethics washing. By pointing out these issues, we aim to highlight the obstacles that hinder translation of Trustworthy medical AI to practice and prevent it from fulfilling its unmet promises.

## Introduction

Worldwide, healthcare systems face overwhelming challenges due to multiple factors such as ageing populations, increasing costs, the recent pandemic, and shortages of labor force (1, 2). A deciding factor to battle this crisis could be digital technology, specifically artificial intelligence (AI) (3). By facilitating efficiency gains and by tackling skilled labor shortages AI could help to significantly reduce the pressure on healthcare systems (4). It therefore holds the groundbreaking capability to improve healthcare delivery, if its potential is fulfilled in a trustworthy and ethical manner.

This seemingly paints a bright future for AI in healthcare: Headlines suggest that AI has already exceeded the performance of human doctors in various medical fields (5, 6). At the same time, so-called “Trustworthy AI” guidelines exist with a collective effort of private and public stakeholders (7) to make AI trustworthy in order to fulfil its potential in healthcare in a safe and ethical way (8–10).

But what is the de facto state of AI in healthcare today? The high hopes and perceived success stand in stark contrast to clinical reality. There are only a limited number of AI tools accessible within the clinical environment (11–13), and as of now, we are not aware of any AI-based healthcare tool being incorporated into clinical guidelines as an established norm of medical practice. And even when readily accessible, the tools do not always align with the associated ethical requirements. An ever-increasing number of AI ethics and “Trustworthy AI” guidelines (8, 14), are alarmingly underutilized (15). There are reports of concerning ethical misconduct, e.g., tech companies collecting more sensitive patient data than they publicly announce (16), algorithms significantly underestimating the risk of illness or illness itself in underserved patient-populations furthering racist discrimination (17, 18) or people being improperly counseled on their eating disorder by an AI-bot without their knowledge or consent (19).

How can we explain the discrepancy between hype and reality, the heightened focus on AI breakthroughs in medicine while so few AI systems are effectively and successfully translated to the clinical setting? In this position paper, we address this question by providing a fresh perspective from an ethical viewpoint. First, we identify two translational failures. We then explain how the lack of an operational definition of “Trustworthy AI” contributes to these failures. Our conclusion is that an operational definition of Trustworthy AI is necessary to have a tangible impact on the translation of AI in healthcare tools into the clinical setting.

### The first translational failure: many exploratory studies, little validation

The attention-grabbing outcomes, often highlighted in the media, are largely centered around what we know as exploratory studies (20). They aim to establish proof-of-concepts (PoCs) but do not encompass the complete process needed to turn these advancements into tangible clinical applications. Exploratory studies are indispensable for pinpointing potential valuable applications for AI in healthcare: Only successful PoCs can provide a rationale, from both an economic and ethical standpoint, for securing funds to support subsequent expensive confirmatory studies (20). Results from exploratory studies must undergo thorough validation via confirmatory studies for the safe transition of exploratory findings into the clinical setting; and they must adhere to all the quality benchmarks typical of studies that establish the evidence of their effectiveness, such as sufficient statistical power and predefined study protocols (20). Considering their cost, they are typically included in a commercial product development process. This progression, starting from the initial PoC stage to the eventual creation of a medical product for use in clinical settings, is referred to as translation (21–23). From this point of view, the scarcity of validated clinically available AI-systems despite a growing number of successful PoCs indicates a massive failure in product development and a lack of validation. This gap between research and availability in clinical practice is referred to as translational gap.

### The second translational failure: the gap between ethical principles and ethical practice

Another type of translation that is often overlooked is the topic of ethics. On one hand, ethical questions surrounding AI are a much-discussed topic in science and beyond. Several meta-analyses summarize hundreds of frameworks and guidelines for AI ethics (23–25), authored by standardization bodies, academia and industry, (supra)national bodies and government organizations (26, 27). It was, however, noted that despite this inflation of guidelines on how to perform AI development ethically, there is no shortage of reports of unethical use of AI (17–19, 28). One reason for this is the very abstract nature and limited practical applicability of these frameworks to researchers and developers of AI systems (28). They are simple principle-based guidelines (29–31). A review found that 75% of major ethical guidelines only provide high-level principles with very little detail, and over 80% offer no or the lowest level of practical insights (32).

Simply put, the existing frameworks focus on “what” to do but do not give any guidance on “how” to do it in practice (33). To date, there are barely binding and concrete regulatory incentives, and even if private organizations choose to implement their product following an ethics guideline, there are no clear instructions on how to do that. For example, there are over 20 fairness metrics to choose from, some of which conflict each other (34). How does one decide which one to use? And how should the sub-groups for the fairness testing be defined? When conducting bias assessments related to ethnicity, which criteria should guide the selection and number of ethnic groups to include in the testing process? These questions remain unanswered when applying only principles. These questions are simply examples of multiple specific challenges someone might face during the operationalization of AI principles. Thus, the reality is that there is a widespread ignorance of ethical considerations in AI development in healthcare and it remains unknown how we should perform the translation of ethical principles to practical development (35). The interpretation, relevance, and implementation of trustworthy AI principles is highly context-dependent and poses an additional challenge (36, 37), and translation of ethical principles into concrete tasks and actions consequently often fails. This is the second translational gap: the failure to transfer ethical principles to practice.

### The unmet promise of “trustworthy AI” due to the lack of an operational definition

Trust and trustworthiness stand as fundamental pillars in AI ethics guidelines and the scientific discourse aimed at fostering ethical development, implementation, and utilization of AI in healthcare. Do these two types of failing translation constitute a crisis of “Trustworthy AI”? Seemingly, the answer is yes, since the dimensions of clinical validation and ethical development have been consistently reported as cornerstones of the idea of “Trustworthy AI”: The European Commission's High-Level Expert Group on Artificial Intelligence (AI HLEG) presented its Ethics Guidelines for Trustworthy AI (38), placing the concept of trustworthiness at the forefront, and putting major emphasis on performance validation and ethical development. Other major international frameworks, e.g., from the OECD have argued in a similar vein (14, 39–42). As described in the preceding sections on the two failures of translation, reality does not live up to these claims.

While we acknowledge this perspective on the crisis of “Trustworthy AI”, we suggest that this cause-and-effect relationship also works the other way around—a direction that is often overlooked. We believe that a crisis of “Trustworthy AI” leads to these translational failures. In other words—the reality does not only fall short of the theoretical claim; rather, the theoretical concept hinders its transfer into reality. We argue that a major cause of the aforementioned challenges is a lack of operationalization what “Trustworthy AI” means in practice. There remains substantial ambiguity within the above-mentioned documents and in the scientific literature as to what exactly trust and trustworthiness mean, and what the requirements are for AI to be considered trustworthy (43).

While some ambiguity and some vagueness are inherent to broad umbrella terms like trustworthiness, what is absent is an operational definition. An operational definition, in essence, provides a precise and specific characterization of a variable or term, typically outlining the procedures or tests employed for its measurement or observation. It places greater emphasis on the observable and measurable aspects, focusing less on defining what something “intrinsically” is and more on the exact observations, procedures, or measurements necessary for its existence (44, 45).

In our context, this does not negate existing high-level or conceptual definitions, e.g., which principles constitute Trustworthy AI. Contrarily, it builds on them but shifts the focus away from principles to the practices that manifest these principles concretely in an AI system. Relating back to the previous simple example, many guidelines agree that fairness is an important principle in Trustworthy AI, but which fairness metrics should or should not be used for AI in healthcare and how might this be different for various applications and contexts? Answering this would be part of an overall operational definition of Trustworthy AI. We see this lack of an operational definition as one of the crucial factors that lead to failure of translation of AI in healthcare. How can trustworthiness of AI in medicine be described, not as an abstract phenomenon, but as a measurable, observable and verifiable property? The lack of an answer to this question does not only hamper the translation of AI, but also poses two major risks. We will outline in the following how the lack of an operational definition leads to those risks, which in turn amplify the translational failures.

### Lack of an operational definition, its risks and how it leads to translational failures

The lack of an operational definition constitutes not only a problem in itself but leads to profound risks in consequence: (a) the unintentional misuse of the term “Trustworthy AI” and (b) the risk of intentional abuse by industry stakeholders. In the following we discuss these points in more detail.

a) Unintentional misuse of the term “Trustworthy AI”

The lack of an operational definition of trustworthiness leaves room for unintentional implicit interpretations and connotations of what Trustworthy AI means. Trust is e.g., often implicitly described as a value in itself (46). The literature, therefore, commonly portrays trust as generally desirable; A scoping review (8) found that guidelines call for various variations of trust: trust in research and technology, trustworthy AI developers and organizations, trustworthy “design principles”, or the importance of customers’ trust. These calls are either not further explained at all or only justified by vague positive pointers like “because overall trust in the recommendations, judgments and uses of AI is indispensable for AI to “fulfil its world changing potential”. The review found only one guideline explicitly warning against excessive trust in AI.

In contrast to this, we can understand trust not as a general goal in itself (47–49). Rather, the value of trust stands and falls with the circumstances under which trust is given and whether these justify the trust. This has to do with the structure of trust—there is always a risk that trust could be betrayed. In this sense, two errors might occur (46): (1) We may deem someone trustworthy who is not, or (2) we may *not* deem someone trustworthy who is. In the case of (1), trusting someone who is not worthy of our trust may lead to exploitation and betrayal. In the case of (2), not trusting someone who is trustworthy may lead to harm for both the would-be trustor and the would-be trustee. The first misses the intrinsic and instrumental values associated with trusting. In the second case there are experiences of negative feelings about not being trusted. Trust can therefore be understood as not an end in itself, but has to be placed in an careful and justified manner.

Therefore, in analogy, the core danger for AI systems is not lack of trust but misplaced trust: not trusting an AI system in the medical field that meets certain—to be defined—criteria, and e.g., makes better medical decisions in certain situations, can have dangerous consequences. At the same time, however, the opposite situation of misplacing trust must be considered: AI healthcare models are often tested under unrealistic clinical conditions (50, 51) and fail at generalization or perform considerably less successful when confronted with new data and clinical reality (51–53). In addition, there is the risk of a substantial publication bias (54)—studies with positive results are more likely to be published then those with negative results. Thus, trust can be harmful if it is placed without reflection in AI systems that are not validated and do not deserve this trust.

In summary, without a clear operational definition, the term “Trustworthy AI” is highly vulnerable to unintentional misuse and implicit interpretations, often wrongly assigning inherent value to trust. This has profound implications for translation. Translation cannot be successful if the primary goal is trust or trustworthiness on its own. Instead, it should be regarded as a tool with a well-defined operational definition of what trust (worthiness) involves, including how it can be effectively implemented and measured.

Additionally, this situation is complicated further by the fact that the debate of Trustworthy AI is inherently inter- and transdisciplinary (55). Next to ethics, the crucial fields span from incentives for technical and commercial standardization and certification (56), to law and policies (57–59). While regulation and certification could serve as instruments in enforcing Trustworthy AI, it requires an operational definition of Trustworthy AI to be executed, As of now, the terms trust and trustworthiness are widely absent in the legal texts concerning regulations (60).

b) Intentional Abuse of the term “Trustworthy AI”

Without an operational definition of trust and trustworthiness we run the real risk that the concept will be diluted and becomes an empty shell. Consequentially, this gives an opportunity for actors with questionable intentions to claim ethical development without any real relevance and action. Thus, there is not only a risk of unintentional misuse of concepts caused by confusion lack of definition, as we have shown under (a), but also of directed and intentional malpractice. The term “ethics washing” refers to such deceptive practice where organizations or companies present a superficial appearance of ethical considerations and values, without implementing substantive ethical practices or ensuring ethical behavior (10). Such practices are used to appease public concerns, giving the impression of responsible conduct, while the criticized practices persist within the organization (8, 61, 62). Even if ethicists are employed within organizations, they can find themselves in situations with significant power imbalances, especially when corporate or financial interests are at play, as exemplified by the dismissal of a renowned ethicist from a major tech corporation (63, 64).

Industry can exploit the lack of an operational definition to have a major impact on policy making (61). Thomas Metzinger, a highly renowned philosopher, was a member of the 52-member High-Level Expert Group on Artificial Intelligence (HLEG AI) that was responsible for drafting the Trustworthy AI guidelines of the EU (65). He was one of only four ethicists alongside 48 non-ethicists from politics, universities, civil society, and mainly industry (62). He reflected on the resulting framework as follows: “As a member of the expert group, I am disappointed with the result that has now been presented. The guidelines are lukewarm, short-sighted, and deliberately vague. They ignore long-term risks, gloss over difficult problems (“explainability”) with rhetoric, violate elementary principles of rationality and pretend to know things that nobody really knows.” (62) Given how much impact this framework already had on the field of “Trustworthy AI”, these insights are more than concerning. According to Metzingers report, there is a considerable danger that the whole concept of “Trustworthy AI” was deliberately kept vague and lacks an operational definition solely to accommodate industry interests (62). We agree with Metzinger when he warns that relying on this framework will lead to problems with tokenism, including conceptual smoke screens and mirrors, highly paid dependent industrial philosophers, self-invented quality seals, and non-validated certificates for “Ethical AI made in Europe”.

It is important to mention that this does not mean that the EU Trustworthy AI guidelines cannot be used for rigorous ethical assessment of healthcare AI. Various frameworks guiding the transfer of ethical principles to practice have been developed (66). The Z-inspection process, for example, is an approach for assessing and improving the trustworthiness of AI systems (36, 37, 67). In essence, it is an exploration of ethical considerations and dilemmas, while adhering to the EU Trustworthy AI guidelines. It involves multidisciplinary teams of experts who evaluate various dimensions of AI systems, including technical robustness, safety, fairness, transparency, and accountability, based on socio-technical scenarios. Another approach, Embedded Ethics, focuses on the integration of ethical principles throughout the entire development process of technologies, often involving incentives to include philosophers as members of AI software development teams (68, 69). While the aforementioned methods pertain to freely available frameworks, there are also commercially-oriented approaches such as Digital Catapult, whose framework also closely adheres to the EU Trustworthy AI guidelines (70). While the mentioned approaches are validated and published incentives to translate ethical principles into practice, there is a genuine risk that actors with questionable intentions piggyback on such successful applications claiming similar success without the same rigor. In these cases, the term Trustworthy AI becomes an empty shell, and a pretext to nurture a false and potentially dangerous sense of security. Ethics should not be reduced to a form of industry self-regulation, but rather integrated as an essential component of technological advancement in healthcare. Without a clear operational definition of what trust(worthiness) is, we will likely not achieve this goal. To prevent the translational failures that we have introduced previously and to achieve proper validation and ethical standards of AI in healthcare, what constitutes Trustworthiness of AI needs to be operationally defined, and not by industry alone.

## Conclusion

In conclusion, we present the position that the translational gaps in healthcare AI significantly result from a lack of an operational definition of (trust)worthiness. This leads to (a) unintentional misunderstandings about the term and (b) creates opportunity for intentional misuse in the form of ethics washing. To prevent these risks and foster genuine trust in AI for healthcare, it is crucial to establish an operational definition of trust(worthiness) which includes guidance on how to tangibly produce, measure and evaluate trustworthiness of AI. This definitory work must be performed carefully since there are many possible conceptualizations of trust(worthiness). For example, whether trust in AI pertains directly to the technology itself or indirectly to the humans associated with it remains a key question. Embracing a socio-technical perspective that considers both AI systems and human stakeholders is crucial for fostering genuine trust and improving the translation of healthcare AI into clinical practice, requiring interdisciplinary collaboration and inclusivity in ethical assessments and development processes. Through the development of an inclusive and clear operationalized definition of Trustworthy AI the concept can evolve from an all too often empty term to an effective ethical practice fulfilling its yet unmet promises.

## Data Availability

The original contributions presented in the study are included in the article/Supplementary Material, further inquiries can be directed to the corresponding author.
